# Improved protein arrays for quantitative systems analysis of the dynamics of signaling pathway interactions

**DOI:** 10.1186/1477-5956-9-53

**Published:** 2011-09-15

**Authors:** Xiaoyu Wang, Ying Dong, Ameena J Jiwani, Yonglong Zou, Johanne Pastor, Makoto Kuro-o, Amyn A Habib, Minzi Ruan, David A Boothman, Chin-Rang Yang

**Affiliations:** 1 Harold C. Simmons Comprehensive Cancer Center, University of Texas Southwestern Medical Center at Dallas, Dallas, TX 75390, USA; 2 Department of Radiation Oncology, University of Texas Southwestern Medical Center at Dallas, Dallas, TX 75390, USA; 3 Department of Pharmacology, University of Texas Southwestern Medical Center at Dallas, Dallas, TX 75390, USA; 4 Department of Pathology, University of Texas Southwestern Medical Center at Dallas, Dallas, TX 75390, USA; 5 Department of Neurology and Neurotherapeutics, University of Texas Southwestern Medical Center at Dallas, TX 75390, USA; 6 North Texas VA Healthcare System, Dallas, TX 75216, USA; 7 VigeneTech, Inc. Carlisle, MA 01741, USA

**Keywords:** protein array, quantum dot, quantitative analysis, signaling pathway, cross-talk, image analysis, glioma, systems biology

## Abstract

An improved version of quantitative protein array platform utilizing linear Quantum dot signaling for systematically measuring protein levels and phosphorylation states is presented. The signals are amplified linearly by a confocal laser Quantum dot scanner resulting in ~1000-fold more sensitivity than traditional Western blots, but are not linear by the enzyme-based amplification. Software is developed to facilitate the quantitative readouts of signaling network activities. Kinetics of EGFRvIII mutant signaling was analyzed to quantify cross-talks between EGFR and other signaling pathways.

## Background

The emerging field of Systems Biology focuses on the most daunting challenges in biology and medicine. It is an attempt to understand how all parts of the cell - genes, proteins, and other molecules - work in concert to create complex living organisms and analyzing how entire biological systems function, both in health and in sickness. Systems biology always relates big amount of data, which makes high throughput technologies are crucial. The rapid advance of high throughput technologies has enabled scientists to broaden their research from detailed investigation of a few selected genes/proteins to global gene/protein expression profiles and network analysis. Among the network analysis, cellular signal transduction networks play an important role in regulating cellular processes, such as proliferation, cell growth and death. Proteins are the work-horses that carry out these functions. Therefore, it is crucial to capture the dynamics of protein kinases and post-translational regulations within cellular signal transduction networks for understanding how the signaling pathways are operated in healthy versus disease conditions.

Reverse phase protein lysate array (RPPA), originally introduced by Drs L. Liotta and E. Petricoin [1], is designed for measuring protein expression in a large number of biological samples quantitatively. Sample lysates were spotted in series of dilutions to generate dilution curves for quantitative measurements. Arrays are probed with a primary antibody followed by a species-specific secondary antibody similar to the Western blot. The detection signal comes from the tag on the secondary antibody. A range of detection tags have been developed including colorimetric, fluorescent, near-infrared (IRDye), and Quantum dot (Qdot) assays [2-6]. RPPA has been applied to protein monitoring for biomarker discovery and/or signal transduction proteins in response to various biological stimuli or chemical treatments [7-10]. However, to use RPPA as a quantification assay is a real challenge, because the linear signals, the foundation of quantification, are difficult to be obtained by using the common enzyme-based (horseradish peroxidase, HRP) signal amplification systems such as Tyramide Signal Amplification (TSA™, Molecular Probes), or Catalyzed Signal Amplification (CSA™, Dako) [2-5]. Non-enzyme based signal detection based on IRDye with Odyssey scanner (LI-COR) [11] as well as Qdot with hyperspectral imaging microscope (not commercial available) [6] have been reported. Here, we report another alternative non-enzyme amplification approach using Qdot and commercial available confocal laser Qdot scanner for protein quantification.

The Qdot is a nano-metal fluorophore with bright and linear signal, and the advantage of using Qdot is it has no photo-bleaching effect that often occurs while using organic fluorophores. In combination of confocal laser Qdot scanner, we present an enhanced version of the RPPA platform for sensitive, reproducible and quantitative cellular signal transduction network measurements. The cell lysis buffer is optimized for RPPA printing and dissolving whole cell proteins without using urea. The thin-coated-nitrocellulose slide is chosen for strong protein binding and low fluorescence background. A confocal laser Qdot scanner is utilized to amplify and maintain the signal linearity. The widely used enzyme-based amplification is not linear, resulting in nonlinearity signals that not suitable for the quantification is also demonstrated. To further reduce background fluorescence from nitrocellulose and increase signal/noise ratios, the advantage of using confocal laser is that it can focus Laser right above the nitrocellulose coating. Integrated software is used to automatically analyze array images, qualify and quantify spots in series, and generate serial dilution curves to determine the relative protein levels and phosphorylation states in the samples.

To demonstrate the capacity of our platform to capture the dynamics of signaling responses, and determine the sensitivity to detect minute changes, glioma cancer cells expressing constitutively activated EGFRvIII mutant under tetracycline control were analyzed by protein arrays. The EGFRvIII mutant is a common oncogenic mutant co-expressed with wild-type EGFR in glioblastoma (GBM) [12]. EGFRvIII is unable to bind ligand and signals constitutively. Kinetics of signaling after conditional induction of EGFRvIII expression was analyzed to quantify the response. The dynamics of pathway interactions (i.e. cross-talks) between EGFR pathways and other signaling pathways were then captured.

## Results and discussion

Understanding complex cellular systems will require the identification and analysis of each of its components and allow determination of how they function together and are regulated. A critical step in this process is to determine the biochemical activities of the proteins and how these activities themselves are controlled and modified by other proteins. Traditionally, the biochemical activities of proteins have been elucidated by studying single molecules, one experiment at a time. This process is not optimal, as it is slow and labor intensive. To obtain a global view of molecular events instead of individual molecules, we utilize the protein array approach to monitor the molecular network response.

### Linear Dynamics for Quantization

**Figure 1:**
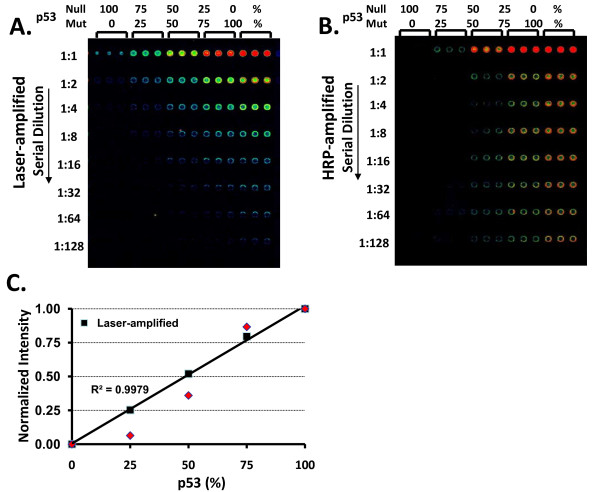
**Linearity of Laser-amplified Qdot-RPPA for quantifying protein levels**. **A**. Human H358 (p53 null) lung cancer cell lysate was mixed with H2009 (p53 mutant) cell lysate proportionally (%) as indicated, then printed with seven serial dilutions in triplicate on protein array. The array was probed for total p53 levels and signals amplified by a confocal laser Qdot scanner. **B**. The same lysates in A., but signals amplified by CSA kit (Dako). **C**. Linear regression curve of 1:8 dilution from Panel A is shown here. X axis is % of p53 mutant lysate in the mixed sample and Y axis is the corresponding Qdot intensity corrected with total protein loading, then subtracted the background signal at the 0% of p53. Black squares is the data from Panel A; red diamond is the data from Panel B. 25% change of p53 can be detected by Laser-amplified Qdot-RPPA as indicated by the black linear regression line. In contrast, linearity was lost with HRP-amplification shown as a red sigmoid curve.

To test the linearity of Qdot signal, we produced cell lysates with artificial gradient of p53 protein by mixing null p53 cell lysate (human lung cancer H358 cells with null p53 gene) and p53 mutant cell lysate (human lung cancer H2009 cells with overexpressing mutant p53) in different proportions. Figure 1A is the array image amplified by confocal Laser scanner. Figure 1B is the array image amplified by CSA (HRP-based) system. As shown in Figure 1C, R2 value of the Laser-amplified linear regression curve is close to 1 indicating Qdot-RPPA can distinguish at least 25% change of protein levels within the linear range, verse the signals of CSA-amplification showing the sigmoid response indicating lost of the linearity.

### Sensitivity

**Figure 2:**
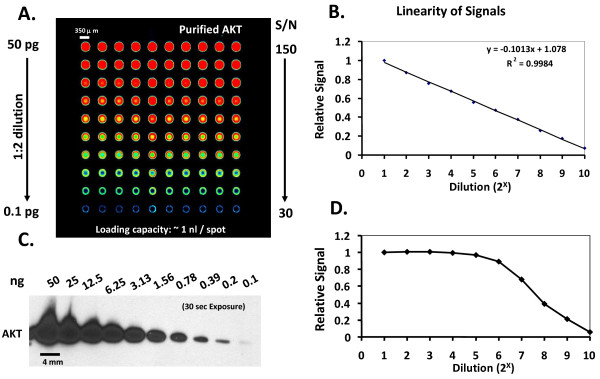
**Sensitivity and linearity of Qdot-RPPA**. **A**. Qdot-RPPA could detect as low as 0.1pg of purified AKT. From top to bottom are 10 serial dilutions. From left to right represent 10 replicates. The Signal/Noise ratios are from 30 to 150 as indicated. **B**. The linearity of the intensity. X-axis indicates the exponent of 2-fold serial dilution and Y-axis represents the relative intensity normalized to the highest concentration. **C**. The corresponding Western blot showing the detection limit is 0.1ng, a 1000× difference in sensitivity. **D**. The signals of the Western blot were quantified from film and graphed as in **B**. The sigmoid curve indicates signal saturation and low dynamic range using Western blot.

In Figure 2, using purified Akt protein, Qdot-RPPA, like CSA-RPPA, can detect as low as 0.1 pg compared to 0.1 ng of the detection limit using traditional Western blot in normal conditions, at least 1000-fold more sensitive (Figure 2A vs. C). Moreover, the signal linearity over serial dilutions makes Qdot-RPPA a reliable tool for quantification (Figure 2B vs. D).

### Specificity

**Figure 3:**
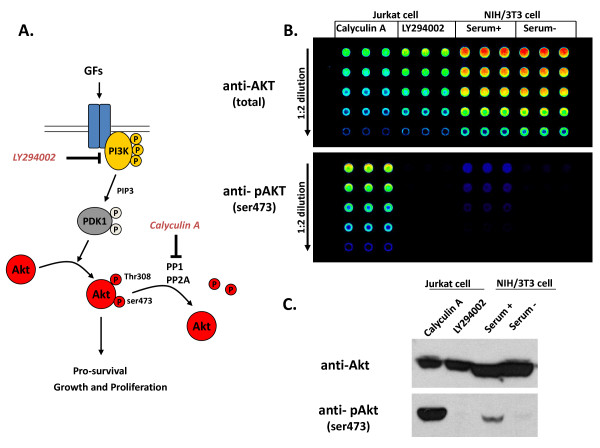
**Specificity of Qdot-RPPA**. **A**. The regulation of AKT signaling pathway. Calyculin A is a phosphotase inhibitor leading to phospho-AKT accumulation. LY294002 inhibits PI3K phosphorylation therefore blocks the activation of downstream AKT signaling pathway. **B**. Cells were treated with inhibitors or serum as indicated to alter AKT activity. RPPA probed with either total AKT or phospho-AKT antibodies. All the samples were spotted in triplicates with 5 serial dilutions. **C**. Corresponding Western blots using the same lysates in **B**.

Qdot-RPPA can detect specific kinase activities with validated phospho-specific antibodies. In Figure 3, Qdot-RPPA distinguishes phospho-AKT (pAKT ser473) activation in the PI3 kinase inhibitor (LY294002) vs. phosphatase inhibitor (Calyculin A) treatments as well as with vs. without serum stimulation. Total AKT is not altered during treatment. Thus, with a pair of validated total and phospho-specific antibodies, Qdot-RPPA can be used to monitor functional status of a given kinase under different treatments, or diseases.

### Reproducibility

**Figure 4:**
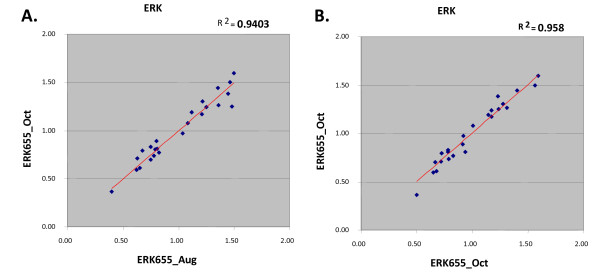
**Reproducibility of Qdot-RPPA**. The same samples were spotted two months apart, and probed with ERK antibody. **A**. The scatter plot and R value of array readouts from two months apart. **B**. The scatter plot and R value of array readouts from the duplicate experiments at the same time.

The signals of Qdot are reliable over time. Examples of ERK protein stain are shown in Figure 4. The same samples were spotted and hybridized with Qdot at the different time in duplicate. R^2 ^values are about 0.95.

### Linearity Tests for Phospho-specific Antibodies and Caspase

**Figure 5:**
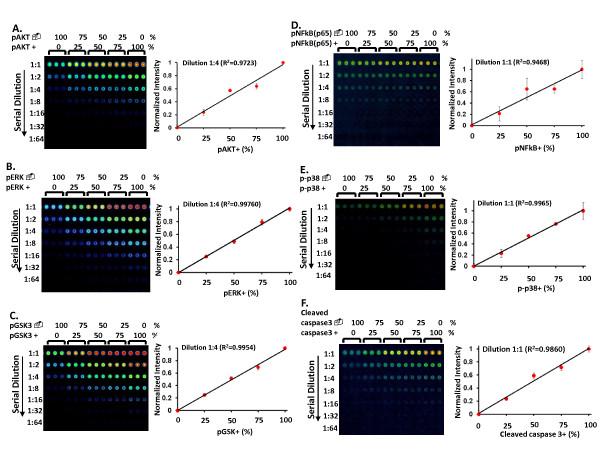
**The Linearity tests for Quantifying Cellular Signaling Kinases**. A series of negative (-) and positive (+) controls for phospho-specific and caspase 3 antibodies were mixed proportionally to generate artificial gradient of target proteins for the linearity tests using laser-amplified Qdot-RPPA. Examples shown here are (**A**) pAKT, (**B**) pERK, (**C**) pGSK3, (**D**) pNFkB, (**E**) pp38, (**F**) cleaved caspase 3. pAKT+, pERK+ and pGSK+ are Jurkat cells treated with phosphatase inhibitor, calyculin A, to accumulate these kinases in phosphorylated states. pNFkB+ is HeLa cells stimulated with TNF (20 ng/ml, 5 min) to activate NFkB. p-p38+ is C6 glioma cells treated with anisomycin to activate p38. Cleaved caspase 3+ is cytochrome c treated Jurkat cell lysates to induce the cleavage of caspase 3. X axis is the gradient (25% intervals) of targeted protein. Y axis is the calibrated signal intensity after subtracting the background signal at the 0% gradient. R^2 ^values of linear regression curves close to 1 indicate laser-amplified Qdot-RPPA can distinguish at least 25% change of protein levels within the linear range. Error bars were derived from the three replicates of each sample.

In Figure 5, a series of commercial available negative and positive controls for indicated phospho-specific and caspase 3 antibodies were mixed proportionally to generate artificial gradients of target proteins: 2A. pAKT; 2B. pERK; 2C. pGSK3; 2D. pNFkB; 2E. pp38; 2F. cleaved caspase 3. The intensities from 0% gradients were considered to be non-specific signals from the antibodies, and deducted from the readouts of other gradients. The Y axis is the calibrated signal intensity after subtracting the non-specific background signal. R^2 ^values of these Laser-amplified linear regression curves are near 1 assuring that Qdot-RPPA can distinguish at least 25% change of protein functional levels while using validated antibodies.

### Array Image Processing and Statistic Readouts

**Figure 6:**
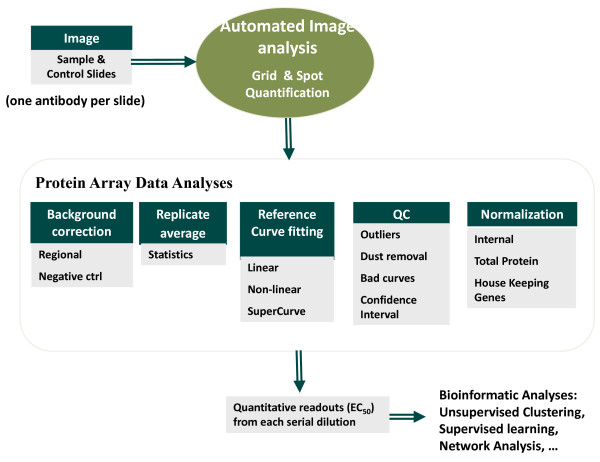
**Software Architecture of MicroVigene™for High Throughput Protein Microarray Analysis**. MicroVigene™ integrates protein array image analysis and statistical signal data analyses in a single automated process. The array images are analyzed in a batch process. Data analyses including background correction, statistics of replicates, dilution curve fitting, quality controls, normalization can be carried out automatically. The final outputs are the EC50 values from each serial dilution arranged in a spread sheet that can be further analyzed by other Bioinformatics tools.

Compared to commercially available DNA microarrays, protein arrays present additional challenges in image analysis. The variety of array formats, spot shapes, and intensity profiles makes it challenging to extract spot signals correctly. In addition, the different array substrates, printing mechanisms and protocols, staining/blocking processes, and broad applications result in various kinds of complex images. It is extremely difficult to develop one algorithm fit all applications. MicroVigene™ is implemented based on the object-oriented technology and enable a robust software system integrating with multiple algorithms that is flexible, configurable as well as extensible to provide customized solutions, support any future needs, and adapt along with this emerging field through proper plug-ins (Figure 6) [13].

**Figure 7:**
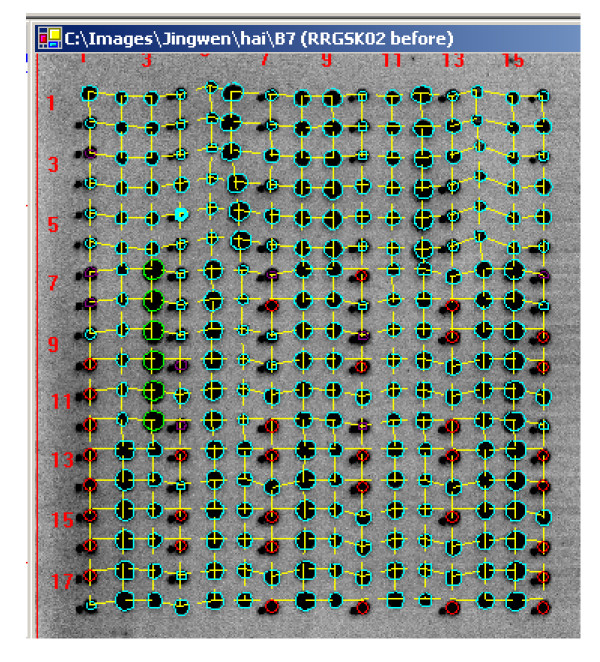
**Actual Boundary and Flexible Grid Algorithms**. Image and spot shifts among arrays are the major challenge for the automation of array image processing. The fixed spot grid cannot be applied to all images. The actual boundary and flexible grid algorithms are designed to make spot boundary and grid flexible enough to compensate the shifts. These algorithms are essential for the successful automation of array image analysis using MicroVigene™.

**Figure 8:**
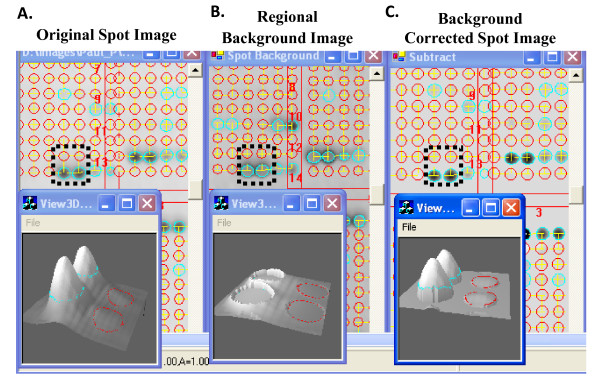
**Regional Background Correction Algorithm**. This algorithm is to correct the original spot signal intensity (**A**) with local background (**B**). The real spot signals (**C**) can be extracted. Four spots within the black dashed square boxes in 2D view are shown in the 3D view windows below. This algorithm improves accuracy, consistency, and sensitivity for image processed using MicroVigene™.

Customized MicroVigene™ microarray image analysis software has been developed for high throughput, automatic array image processing and quantitative readouts from serial dilutions of samples. The software implements the actual boundary algorithm for spot identification/segmentation that is resistant to the spot shift and image shift (Figure 7); the regional background algorithm for local non-uniform background correction and sensitive spot quantification (Figure 8).

**Figure 9:**
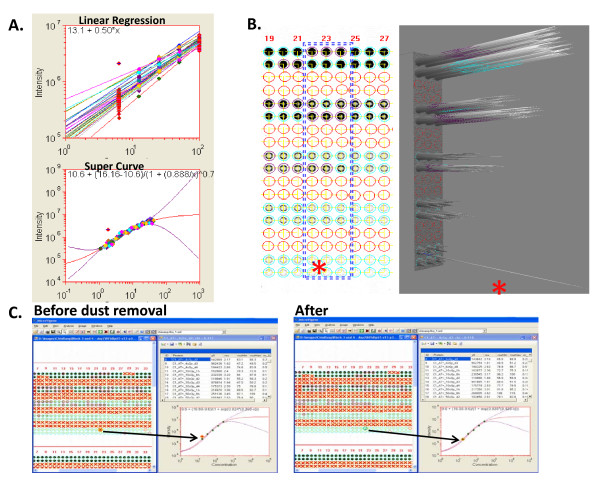
**Automation of Qdot-RPPA Image and Data Analysis**. Several features of MicroVigene™ software are to facilitate the automatic batch process. **A**. Super Curve: Instead of generating multiple linear regression curves for each serial dilution, the Super Curve algorithm uses all spots on the same array to fit one antigen-antibody reaction curve (called Super Curve, the red line) for the quantitative readouts. The purple lines indicate error boundary. **B**. 2D and 3D views of the actual spot boundary algorithm for actual spot identification and dust finding (indicated by asterisk); the total intensity within the defined spot boundary (i.e. volume) was used for Super Curve fitting. **C**. Screen shots of the dust removal feature. Left: before removing the dust, the spot is flagged as an outlier (the red spot pointed by the arrow). Right: after dust removal, the spot fits the Super Curve.

Also, instead of generating multiple linear regression curves for data quantification over each series of serial dilutions, MicroVigene™ implements the SuperCurve algorithm (details in the Methods section) [14] that using all spots within one array to form a sigmoid antigen-antibody binding kinetic curve (i.e. SuperCurve) (Figure 9A). The advantage of SuperCurve is the resistance to the experimental outliers or missing spots on the array compared to regular linear regression curves formed by only a few spots over a serial dilution. The SuperCurve is a consensus curve supported by all spots on the array. This process has been implemented into the automatic data analysis workflow after the spot identification to automatically generate a reaction curve for each antibody as well as the quantitative readouts from each dilution series.

The automatic dust finding and removal algorithm is also implemented to increase the accuracy of curve fitting and the intensity readouts. In Figure 9B, a small but intensified dust spot was automatically detected in the lowest dilution spot, and then visualized by 3D image manually. Without the dust removal, the spot was flagged as an outlier of the SuperCurve verse, after removing the dust signal, the spot fitted in the SuperCurve well (Figure 9C).

In summary, these unique features of MicroVigene™ make it capable of handling shifted and noisy protein array images and enabling the hand-free batch process required for high throughput protein array image processing.

### The Dynamics of EGFRvIII Signaling Network

**Figure 10:**
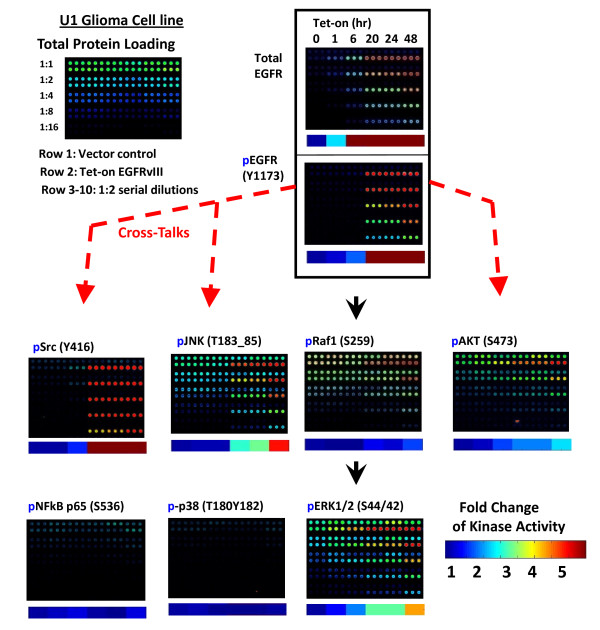
**Signaling network up-regulated by constitutively activated EGFRvIII mutation**. U87MG glioma cells were transfected with a conditional inducible tetracycline (tet-on) sytem. A EGFRvIII expressing line (row 2) as well as vector control (row 1) was treated with tetracycline (1 ug/ml) for 0, 1, 6, 20, 24 and 48 hours as indicated. Activities of signaling kinases were monitored by Qdot-RPPA. Each sample was serially diluted 1:1 to 1:16 for quantification and spotted in triplicate on the arrays, then probed with total and phospho-specific kinase antibodies. Relative fold changes to time 0 were quantified and illustrated with color under each corresponding array images. Black arrows indicate known canonical signaling pathways; Red dashed arrows indicate the cross-talks between Akt, Src and JNK pathways. p38 and NFkB stay inactive.

Effective targeted treatment of glioblastoma (GBM) may require a coordinated inhibition of multiple signals. Much remains to be learned about functional interactions among signal transduction networks in GBM. For example, GBMs express multiple receptor tyrosine kinase (RTK) families and ligands. Increasing evidence suggest extensive cross-talking between RTK signaling networks that have functional implications for multi-target treatment. EGFRvIII is a common oncogenic mutant that is co-expressed with the wild-type EGFR in GBM. We applied Qdot-RPPA to investigate key protein regulators and kinases (total 61 antibodies listed Table 1) that are altered by conditional inducible EGFRvIII in U87MG glioma cell line. The dynamics of pathway interactions (i.e. cross-talks) among canonical EGFR pathway, Akt, Src and JNK pathways after turning on the EGFR vIII were captured (Figure 10). Qdot-RPPA detected the total EGFR, pEGFR and pERK levels changes within one to six hours after adding tetracycline; Activation of Akt [15,16], Src [17,18] and JNK [19-21] happened at later time (20-24 hrs), and, interestingly, pSrc (52.8×) and pJNK (4.4×) were activated at much higher levels than the downstream pERK (3.8×) in the canonical pathway (Array images shown in Figure 10 and fold change in activity shown in Table 2). The results suggest the selection of potential candidates for the future multi-target treatment in GBM, e.g. co-targeting EGFRvIII and Src/JNK kinases for cancer treatment [17,18] since Src and JNK activities are elevated much higher than ERK over time.

**Table 1:** Current Available Validated Antibodies for Protein Kinases and Regulators

AKT	Rb	NFkBp65
pAKT(S473)	pRb(S807_811)	pNFkBp65(S536)
pAKT(T308)	p16	IKBa
ERK1/2	p21	pIKBa(S32)
pERK1/2(S44/42)	p27	MDM2
GSK3ab	CyclinB1	pMDM2(S166)
pGSK3ab(S21/9)	CyclinD3	E-Cadherin
IRS1	Cdk4	Vimentin
pIRS1(Y896)	EGFR	b-Catenin
pIRS1(Y1179)	pEGFR(Y1173)	gH2AX
mTOR	IGF1R	Hsp27
pmTOR(S2448)	pIGF1R(Y1158_1162)	NQO1
PTEN	pIGF1R(Y1162_1163)	sClu
pPTEN(S380)	Raf1	b-Actin
p38	pRaf1(S259)	Smad3
pp38(T180_Y182)	Bcl2	pSmad3(S423/425)
Src	pBcl-2(S70)	Cleaved Caspase 3
pSrc(Y416)	JNK	Cleaved Caspase 8
pSrc(Y527)	pJNK(T183_185)	Cleaved Caspase 9
Stat3	p53	
pStat3(S727)	pp53(S15)	

**Table 2:** Fold Change of Signaling Kinases Up-regulated by Constitutively Activated EGFRvIII Mutation over Time

*Time*	*EGFR*	*pEGFR(Y1173)*	*pRaf1*	*pErk*	*pAkt*	*pSrc(Y416)*	*pJNK*	*pp38*	*pNF-kBp65*
0 hr	1.00	1.00	1.00	1.00	1.00	1.00	1.00	1.00	1.00
1 hr	2.28	1.14	0.88	1.36	1.08	0.99	1.02	1.01	1.08
6 hr	7.31	1.75	0.89	1.89	1.62	1.51	1.04	0.91	1.24
20 hr	23.19	26.19	1.32	2.76	1.88	31.35	2.61	0.93	0.95
24 hr	22.14	25.11	1.17	2.77	1.88	34.39	2.82	0.88	0.92
48 hr	26.39	35.62	1.59	3.79	2.26	52.79	4.37	0.99	1.26

## Conclusions

In conclusion, we have established a quantitative proteomic technology - Qdot-RPPA platform, in conjunction with the emerging Quantum dot (Qdot) nanotechnology, a confocal laser scanner, computer automated array image analysis to generate quantitative outputs. This platform eliminates the signal non-linearity causing by enzyme-amplification, while still keeps the advantages of RPPA such as: minute sample per spot(~1nl); high sensitivity (~0.1 pg); high specificity with validated antibodies; high reproducibility; and large capacity of spots on one slide allowing investigating a large number of experimental conditions in parallel within one array.

The primary contribution of our Qdot-RPPA platform is to take advantage of Qdot linear signal with no photo-bleaching and large dynamic range for quantification by utilizing confocal Laser Qdot scanner. This technology not only offers us the capacity to quantitatively monitor the time series and dose responses of cellular response over signaling network after treatments or different disease stages, but also facilitates the complex functional analysis among different signaling pathways. We also found that thin coated (10 um) nitrocellulose slide with scanning Laser focus above the coating yields low fluorescence background and increases signal/noise ratio.

We have applied Qdot-RPPA platform to study the Dynamics of EGFRvIII Signaling Network with 61 antibodies. The results demonstrated this platform worked well to reveal not only the dynamics of canonical pathway but also cross-talks among pathways. The ultimate goal is to extend this platform with more validated antibodies to provide high sample throughput functional protein data to compliment DNA based genomics array data for systems biology analyses, and provide direction for more in-depth experimentation prediction and hypothesis generation.

## Methods

### Cell Lysate Preparation

p53 control cell lysates were prepared from human H358 (p53 null) lung cancer cells and H2009 (p53 mutant) cells. The rest of control cell extracts were purchased from Cell Signaling Technology: Akt (cat# 9273), p38 MAP kinase (cat# 9213), NF-κB (cat# 9243), and caspase-3 (cat# 9663). Purified Akt protein was purchased from Invitrogen (cat# P2999). U87MG glioma cells were stably transfected with an EGFRvIII mutant using a tetracycline inducible system as described previously [22]. Cells were treated with tetracycline (1 ug/ml), and then, harvested at 0, 6, 20, 24 and 48 hours, respectively. The following cell lysis protocol is optimized to harvest total cell proteins including hard-to-dissolved membrane proteins without using Urea. Cells were washed twice in cold phosphate buffered saline (PBS) and subsequently lysed in 250-350 μL hot lysis buffer (2% SDS; 0.06 M Tris-Cl, pH 6.8; 5% Glycerol). Proteinase inhibitor (Sigma, cat# P8340) and phosphotase inhibitor cocktails (Santa Cruz Biotechnology, sc-45044 and sc-45045) and 2% β-mercaptoethanol were freshly added before use. The cellular lysates were boiled for 5 min on heat block, followed by 1 min vortexing, and then centrifuged at 13,000 rpm for 7 min at 4°C. Cells should be completely dissolved without much precipitates left at this step. Supernatants were transferred to new tubes and stored at -20°C. Protein concentration was measured using Bradford assay (Bio-Rad, cat# 500-0006). All the samples were adjusted to 0.5 ug/uL as the highest concentration on the arrays. However, to correct for protein loading, Sypro Ruby™ protein stain signals were used as described below.

### Qdot-Reverse-Phase Protein Microarray (Qdot-RPPA)

Protein lysates were filtered through 96-well filter plate with 25 μm pore membrane (Phenix Research Products, cat# MPF-009) by centrifuge to remove sticky aggregates, and serially diluted (1:2) 4 times using 1 × lysis buffer. Lysates were arrayed on ONCYTE^® ^AVID nitrocellulose film slides (Grace Bio-Labs, cat# 305170) using a SpotArray™24 Microarray printing system (PerkinElmer) with 55-60% humidity. Spots were separated with 350 μm space in between. Approximately 1 nl of lysate per spot was arrayed using 4 spotting pins (TeleChem/ArrayIt, cat# 946MP3). Slides were dried at RT for about 30 min, and then stored at 4°C. Immuno-staining was performed within one week. We have tested various slides from different vendors. The AVID slides were chosen due to its high protein binding capacity with less protein lost, and thin-coated (10 micron) nitrocellulose film with less auto-fluorescence background, suitable for both Qdot™ and SyproRuby™ stains.

### Laser-amplified Qdot-RPPA and Immuno-staining

The slides were placed into four-chamber plates (ISC Bioexpress, cat# T-2896-1) and incubated at room temperature in Re-blot Plus Mild Solution (CHEMICON, cat# 2502) for no more than 7 minutes to relax protein structure. After the Re-blot was removed, the slides were washed 5 min three times in TBS-T buffer (2.42 g Tris-HCl, 16 g NaCl in 1 L dH_2_O, 0.1% Tween-100, pH 7.6). The slides were incubated in Sea Block blocking buffer (Thermo, cat# 37527) at 4°C overnight, and then blocked with Avidin and Biotin (Dako, Biotin blocking system cat# X0590) sequentially. Between steps, slides were washed with TBS-T buffer. After the blocking, the slides were incubated with primary antibodies (diluted in antibody diluent buffer, Dako, cat# S3022) at 4°C overnight. We used 61 validated primary antibodies listed in the Table 1 and the Additional file 1; validation information is in [3]. Next, slides were washed with TBS-T buffer, and incubated with biotinylated secondary antibodies (Vector, anti-rabbit BA-1000 or anti-mouse BA-9200 IgG) (1:5000) for 30 min, then with Qdot 655-streptavidin conjugate (Invitrogen, cat# Q10121MP) for 30 min. Qdot were diluted 1:200 in boric acid buffer (50 mM Borate, 2% BSA, pH 8.3). The slides were washed with TBS-T buffer 3 times, and MilliQ water once and then briefly spun at 2000 rpm for 5 min to dry the slides. To detect Qdot signals, slides were scanned with full Laser power on a ProScanArray Microarray Scanner (PerkinElmer), and the Laser was focused 10 micron above the slide surface. The excitation wavelength was set at 488 nm and emission wavelength was set at 655 nm using a Qdot 655 filter. Images were saved in 16-bit TIFF format and the maximum signal intensity was 65535.

### Enzyme-based Qdot-RPPA

Qdot-RPPA using the Tyramide and horseradish peroxidase (HRP) amplification system (Dako CSA™ system, cat# K1500) to amplify the Qdot signal was previously described [5,6]. This protocol was performed for the comparison shown in Figure 1B.

### Automatic Array Image analysis and Quantification

Scanned TIFF images were batch-analyzed using MicroVigene™ software (VigeneTech inc. http://www.vigenetech.com/). In RPPA, cell lysates were printed on the arrays. Each array is probed with one specific primary antibody and scanned for one image. It is necessary to have computer-aid image analysis that can process array images automatically to increase the throughput of the number of antibodies one can apply. This work was done in collaboration with VigeneTech, Inc., a leader of developing automated image analysis technology. MicroVigene™ provided unique software customized to analyze the Qdot-RPPA platform. Unique features of the software provide accuracy, sensitivity, and reliable results of automation [13] including flexible grid and actual spot boundary algorithm to quantify spot signals accurately; dust removal algorithm to remove the contaminated signals; regional background algorithm for local non-uniform background correction and sensitive spot quantification. Ten thousand spots can be processed in less than one minute and, with the feature of hands-free batch processing, MicroVigene™ enables the high throughput protein array image analysis. 3D visualization of processed images is also available for manually quality assurance.

Each sample has five dilutions and was printed in triplicate on the array. Therefore, each dilution series has total of 15 data points to minimize errors and increase the confidence of curve-fitting. Instead of generating multiple linear regression curves for data quantification over each series of serial dilutions, MicroVigene implements the SuperCurve algorithm (a 4-parameter logistic-log model, i.e. parameters a-d shown in equation below) that uses all spots within one array to form a sigmoid antigen-antibody binding kinetic curve (i.e. SuperCurve) [14].

$$Y=a+\frac{\left(b-a\right)}{\left(1+e^{\left(c*\left(d-ln\left(x\right)\right)\right)}\right)}$$

where x is the dilution factor and Y is the signal intensity. The signal readout of each dilution series is the intensity of EC50 from the fitted SuperCurve. The assumption is that the same antibody-antigen binding kinetics is taking place at each sample spot, even in the different samples, thus by taking all spots on an array to fit a common response curve can increase the confidence of the curve fitting.

Calibration for protein loading was based on total protein per spot. For estimation of total protein amounts, randomly selected arrays were stained with Sypro Ruby™ (Invitrogen, cat# S11791), and visualized on ProScanArray Scanner (PerkinElmer) with excitation wavelength at 450 nm and emission wavelength at 610 nm. The corrected values were calculated by dividing the EC50 readouts of the antibody to the corresponding EC50 readouts of the Sypro Ruby™ stain. In Figure 5, the 0% positive gradient on the left was considered as a non-specific signal from the antibody, and deducted from the readouts of other gradients. In Figure 10, the intensity of time point 0 was treated as a baseline and relative fold change of activity at the other time points were compared to time 0.

## List of abbreviations

CSA: Catalyzed Signal Amplification; GBM: glioblastoma; HRP: horseradish peroxidase; Qdot: Quantum dot; RPPA: Reverse phase protein lysate array; RTK: receptor tyrosine kinase; TSA: Tyramide Signal Amplification.

## Competing interests

M.R. serves as CEO of VigeneTech, inc. In this study, the MicroVigene software was provided by M.R. based on collaboration. Other authors declare no competing interests

## Authors' contributions

XW carried out the array experiments, protocol development, array image and data analysis, figures and manuscript preparation. YD, JP, MK participated in the early development of the protein array technology, provide personal expertise, protocols, and hand-on training to XW. YZ developed and test cell lysis buffers suitable for protein array printing. AJ, AH developed and provided EGFRvIII cell lines for protein array analyses.

MR carried out software development to integrate the array image and data analyses. DB and CY conceived of overall experimental design, coordination among research groups, and manuscript preparation. All authors read and approved the final manuscript.

## Supplementary Material

Additional file 1 **Antibodies used for Qdot-Reverse Phase Protein Array**. Detail information of antibodies used. Click here for file
